# Efficacy of non-pharmacological interventions for depression in individuals with Parkinson's disease: A systematic review and network meta-analysis

**DOI:** 10.3389/fnagi.2022.1050715

**Published:** 2022-11-10

**Authors:** Yuxin Wang, Xue Sun, Fei Li, Qi Li, Yi Jin

**Affiliations:** ^1^Graduate School, Tianjin University of Traditional Chinese Medicine, Tianjin, China; ^2^Nursing Department, Beijing Tiantan Hospital, Capital Medical University, Beijing, China; ^3^Clinical College of Neurology, Neurosurgery and Neurorehabilitation, Tianjin Medical University, Tianjin, China; ^4^Department of Nursing, Tianjin Huanhu Hospital, Tianjin, China

**Keywords:** non-pharmacological interventions, depression, network meta-analysis, Parkinson's disease (PD), randomized controlled trials

## Abstract

**Background:**

Depression in Parkinson's disease (PD) is a major health concern worldwide. Recently, an increasing number of non-pharmacological interventions have been used in PD to alleviate depressive symptoms. However, it is uncertain which intervention is the best, and related evidence is limited. This network meta-analysis was performed to compare and rank non-pharmacological interventions for PD and analyze their effects on depression to provide evidence for clinicians to choose appropriate non-pharmacological management options.

**Methods:**

The PubMed, Embase, Cochrane Central Register of Controlled Trials (CENTRAL), PsycINFO, China National Knowledge Infrastructure (CNKI), and Wanfang databases were searched from inception to April 7, 2022. Two authors screened all studies, extracted the data, and evaluated the methodological quality. STATA software version 16.0 was used to conduct the network meta-analysis.

**Results:**

Our network meta-analysis included 62 studies involving 3,050 participants and 35 non-pharmacological interventions. Although most non-pharmacological interventions showed non-significant effects, the surface under the cumulative ranking curve (SUCRA) values indicated that the best non-pharmacological intervention for depression was dance (82.3%), followed by LSVT-BIG therapy (77.4%), and CBT (73.6%).

**Conclusion:**

Dance can be considered as an effective therapy for improving depression in patients with PD. In the future, more strictly designed trials are needed to verify the conclusions of this network meta-analysis.

## Introduction

Parkinson's disease (PD), the second most common neurodegenerative disease, is a chronic senile disease (Hirtz et al., 2007). The prevalence of PD increases with age and affects 1% of individuals older than 60 years (Tysnes and Storstein, 2017). PD is characterized by dyskinesia; however, the non-motor symptoms (NMS) of PD have gradually attracted more attention from researchers over the past 10–20 years (Garcia-Ruiz et al., 2014). Neuropsychiatric disturbances and cognitive impairment are the main features of NMS in PD patients (Zhang et al., 2020), while depression is the most common psychiatric symptom. The prevalence of clinically significant depression in patients with PD is reported to be 40–50% (Reijnders et al., 2008). Depression often increases the incidence of disability and dysfunction in PD patients; in addition, it can affect patients' quality of life and the burdens of their caregivers. Moreover, a study by Wu et al. indicated that PD patients with depression had a higher incidence of dementia (Wu et al., 2018). Therefore, early detection and appropriate intervention are extremely important. Currently, the clinical management of depressive disorders in PD includes pharmacological and non-pharmacological treatments. However, studies on the tolerability, safety, and efficacy of antidepressant drugs in PD patients are limited (Assogna et al., 2020). Additionally, pharmacological treatments with side effects can exacerbate the motor symptoms and NMS of PD patients and lead to complications (Uhrbrand et al., 2015; Deuel and Seeberger, 2020).

Owing to the limitations of pharmacological treatments, non-pharmacological treatments have been developed and have gradually gained popularity. Many non-pharmacological treatments have been used to relieve depressive symptoms in patients with PD, and these can be roughly categorized into complementary therapies (e.g., yoga, acupuncture, auricular pressure, massage, music therapy, and dance therapy), traditional Chinese exercises (e.g., tai chi and qigong), physical exercise (e.g., aerobic exercise, resistance exercise, and balance training), virtual reality, cognitive behavioral therapy (CBT), psychotherapy, cognitive training (CT), bright light therapy (BLT), deep brain stimulation (DBS), transcranial magnetic stimulation (TMS), and transcranial direct current stimulation (tDCS) (Jin et al., 2019; Zhang et al., 2019; Deuel and Seeberger, 2020; Triegaardt et al., 2020; Chen et al., 2021; Hong et al., 2021; Huang et al., 2021). Previous studies (Troeung et al., 2014; Ryan et al., 2019; Assogna et al., 2020) have paid more attention to the effects of CBT and TMS on depression in PD patients and indicated that these two therapies are supportive for improving depression. The efficacy of other non-pharmacological treatments for depression in patients with PD remains controversial.

Previous systematic reviews have evaluated the effects of various non-pharmacological interventions on depression in individuals with PD (Cusso et al., 2016; Jin et al., 2019; Hai-Jiao et al., 2020; Triegaardt et al., 2020; Cartmill et al., 2021; Hong et al., 2021; Huang et al., 2021; Takamiya et al., 2021). However, some reviews included non-randomized controlled trials (RCTs) or lacked quantitative analyses (Cusso et al., 2016; Triegaardt et al., 2020; Cartmill et al., 2021; Takamiya et al., 2021); thus, these systematic reviews did not provide strong evidence. In addition, most RCTs compared non-pharmacological interventions with placebo, waiting list, or usual treatments, and only a few RCTs compared two different non-pharmacological interventions (Modugno et al., 2010; Kalbe et al., 2020; Schmidt et al., 2021). To our knowledge, only one systematic review has reported the efficacy of non-pharmacological interventions on depression in PD subjects (Chen et al., 2021). In that study, a population with idiopathic PD was selected, and the interventions included repetitive TMS (rTMS) and CBT. The review also included limited interventions and small sample sizes and excluded some patients with PD. Consequently, a systematic review evaluating the effects of different non-pharmacological interventions and the exploration of more effective interventions is required.

Network meta-analysis (NMA) is a general technique for comparing several interventions simultaneously (e.g., A vs. B, B vs. C) (Lu and Ades, 2004). NMA can compare multiple interventions by incorporating direct and indirect comparisons to select the best intervention based on the relative effects of different interventions from a network of evidence (Catalá-López et al., 2014). Therefore, we performed this systematic review with a NMA of RCTs to provide further evidence to clinicians when choosing appropriate non-pharmacological management options.

## Methods

### Search strategy

In accordance with the Preferred Reporting Items for Systematic Reviews and Meta-Analyses extension statement for reporting systematic reviews incorporating network meta-analyses of health care interventions (Hutton et al., 2015), we searched for randomized controlled trials (RCTs) from inception to April 7, 2022 in the following databases: PubMed, Embase, the Cochrane Central Register of Controlled Trials (CENTRAL), PsycINFO, China National Knowledge Infrastructure (CNKI), and Wanfang. A combination of Medical Subject Headings (MeSH terms or Emtree terms) and free words related to PD, non-pharmacological interventions, depression, and RCTs was used, including: (1) Parkinson's Disease, Parkinson, Idiopathic Parkinson's Disease, Lewy Body Parkinson's Disease, Parkinson's Disease, Idiopathic Parkinson's Disease, Lewy Body Parkinson's Disease, Primary Parkinsonism, Paralysis Agitans; (2) TMS, tDCS, DBS, tai chi, qigong, acupuncture, massage, song, music, dance, aromatherapy, moxibustion, exercise, CBT, psychotherapy, cognitive training, electroconvulsive therapy, transcranial magnetic stimulation, transcranial Direct Current Stimulation, cognitive behavior training, deep brain stimulation, treatment, intervention, therapy, management, rehabilitation, non-pharmacolog^*^, non-pharmacological; (3) depression, depressive symptoms, depressive symptom, emotional depression, depress^*^, central depression, clinical depression, depressive disease, depressive disorder, depressive episode, depressive illness, depressive personality disorder, depressive state, depressive syndrome, mental depression, and parental depression; and (4) randomized controlled trial, randomized, placebo. MeSH and free words were linked by “OR” in each group and searched by “AND” to link each group. In addition, we retrieved data from the U.S. National Library of Medicine Clinical Trial Registry Platform and the Chinese Clinical Trial Registry Platform for trials in progress or ready for publication. The gray literature was also considered in the search. The reference lists of the included literature and related articles were also manually searched to identify eligible studies. The search strategies for all databases are listed in Supplementary Data Sheet 1.

### Eligible criteria

Eligible studies met the following criteria:

(1) Population: Adults (>18 years) diagnosed with PD according to sex, Hoehn and Yahr stage, or disease duration with no restrictions. All participants in the intervention and control groups who were stably taking antidepressants and/or anti-parkinsonian medications were also eligible.
(2) Intervention: Participants in the experimental groups received non-pharmacological interventions with no limits in frequency, duration, style, period, form, or setting.
(3) Comparison: Participants in the control groups received placebo, waiting list, or treatment as usual (TAU) options including usual care, treatment, supportive instruction, or other non-pharmacological interventions that differed from the experimental group. In our study, supportive instruction refers to simple and common advice and tests (e.g., health education, beneficial advice, “classic” game mode) provided to subjects in the control group that differ from professional psychotherapy or psychological instructions. However, original trials comparing only different approaches of the same intervention were excluded.
(4) Outcome: The primary outcome was depression, as assessed by applying validated scales.
(5) Study type: RCTs were included with no limitations in terms of language, country, and type of article (conference papers, abstracts, master theses/doctoral dissertations, and study protocols were permissible; however, reviews were excluded).

### Data extraction and quality assessment

Two authors (YW, XS) independently extracted data including the first author, country, year, sample size, baseline characteristics of participants, duration of disease, Hoehn–Yahr stage, intervention details (type, frequency, and duration), comparison, and outcomes based on a predesigned form within Microsoft Excel. The methodological quality of the eligible studies was independently assessed using the Cochrane risk of bias (RoB 2.0) tool (Sterne et al., 2019) by two authors (YW, XS). The scale consists of five domains: the randomization process, deviations from the intended interventions, missing outcome data, outcome measurement, and selection of the reported result. In terms of the domain algorithm, the risk of bias for each domain was rated as low risk, some concerns, or high risk. If the assessment of risk bias in all domains was “low risk,” then the overall risk bias was considered as “low risk”; if the assessment of risk bias indicated “some concerns” in some domains and there was no “high risk” result in any domain, the overall risk bias was “some concerns”; if the assessed risk of bias was “high risk” in at least one domain, the overall risk bias was “high risk.” The results of the data extraction and quality assessment were cross-checked, and the divergences were resolved through discussion with a third author (YJ). To comprehensively compare the effects of non-pharmacological interventions on depression in PD subjects, we did not exclude medium- and low-quality studies.

### Statistical analysis

The network analysis was conducted using STATA 16.0 (StataCorp, College Station, TX) and a frequentist framework with a random-effects model. For all eligible trials, post-intervention measurements were selected for comparison. Continuous variables were analyzed using standardized mean differences (SMD) with 95% percentile intervals, and the significance was set at α = 0.05. We examined the global consistency and used the node-split model to determine the local consistency. *P* > 0.05 indicated no significant inconsistency between direct and indirect comparisons, and in these cases, the consistency model was adopted; otherwise, the inconsistency model was used. In addition, the inconsistency of closed loops was evaluated using a loop-specific method, and a 95% confidence interval (CI) of 0 indicated no significant loop inconsistency. Each arm was included for comparison, and to discriminate the consistency between the two-arm and three-arm trials, a league table was used to perform the pairwise analysis. The league table was used to analyze the results of the comparisons among the different interventions based on a NMA. To explore the best evidence for improving depression, the SUCRA was used to summarize the ranking probability values. Funnel plots were used to visually evaluate publication bias based on the symmetry criterion (Shim et al., 2017).

## Results

### Study selection

**Figure 1:**
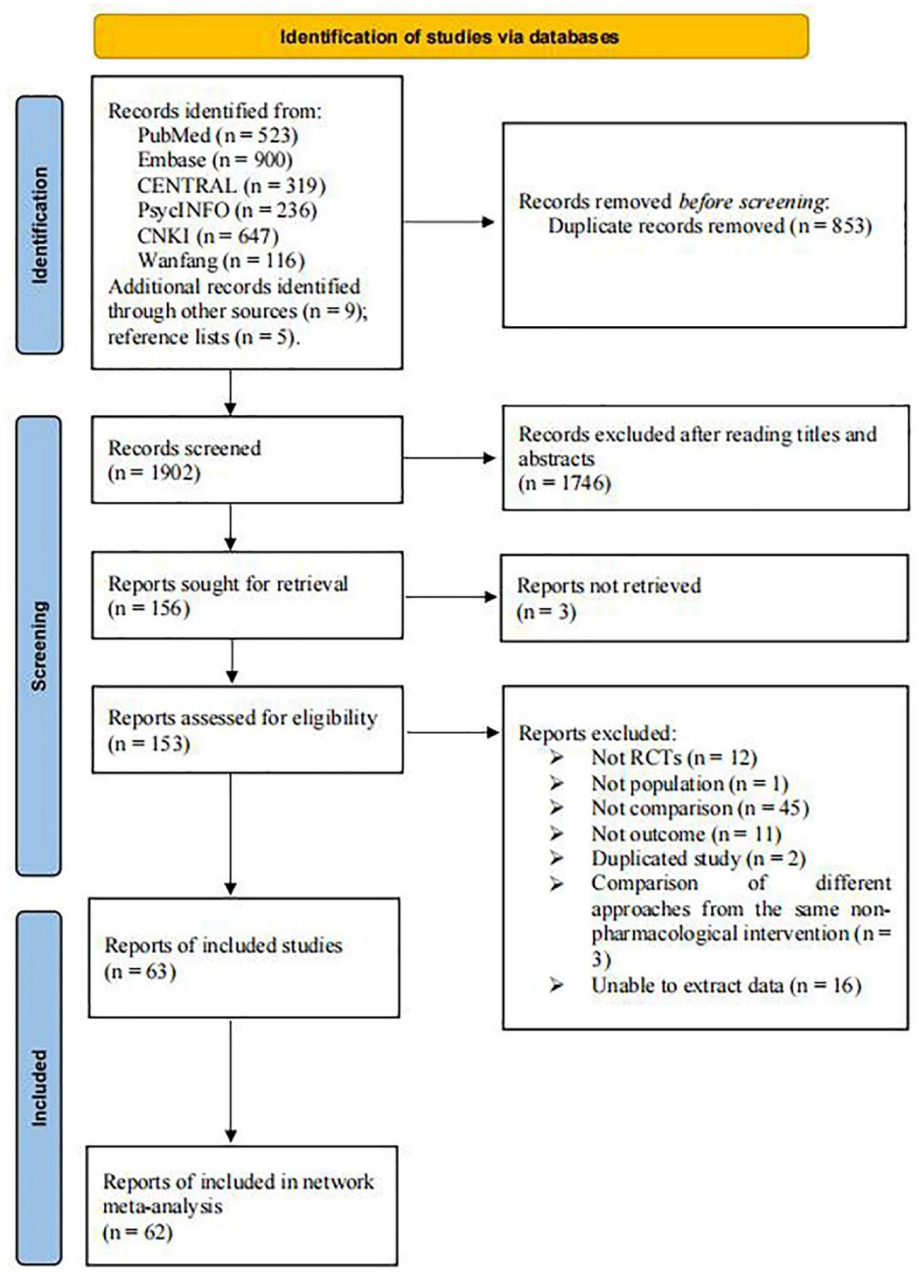
The process of selection of the eligible studies.

A total of 2,755 studies were identified from six databases, while 9 studies were selected from other sources. Five trials were retrieved by screening the reference lists of the included studies and related articles. After removing 853 duplicates, 1,746 articles were excluded after screening their titles and abstracts. Subsequently, 156 articles were retrieved, 153 full-text articles were evaluated, and 63 were included in the systematic review. However, one study did not meet the quantitative analysis criteria of the NMA (Manenti et al., 2018); thus, only 62 eligible RCTs were included in the NMA. Figure 1 demonstrates the process of the literature search and study selection.

### Study characteristics

Table 1 shows the characteristics of 62 eligible RCTs (Chinese = 6, English = 56) published from 2002 to 2021 and involving 3,050 participants. In this NMA, non-pharmacological interventions included a variety of interventions such as BLT (*n* = 4), CBT (*n* = 9), dance (*n* = 3), massage (*n* = 3), music therapy (*n* = 1), DBS (*n* = 1), aerobic exercise (*n* = 2), resistance exercise (*n* = 5), balance training (*n* = 2), mindfulness intervention (*n* = 2), TMS (*n* = 9) including rTMS and rhythmic TMS, traditional Chinese exercise (TCE) (*n* = 5) including Qigong and Tai chi, tDCS (*n* = 1), virtual reality (VR) (*n* = 2), yoga (*n* = 3), CT (*n* = 7), psychotherapy (*n* = 3), acupuncture (*n* = 1), multidisciplinary rehabilitation (*n* = 1), and auricular pressure (*n* = 1). The comparison mainly consisted of placebo, waitlist, TAU including usual care and usual treatment, stretching exercises, supportive instruction (e.g., health education, sleep hygiene advice, regular social interactions, and active testing), and physiotherapy. Among all eligible studies, 60 RCTs were two-arm trials (Wade et al., 2003; Craig et al., 2006; Paus et al., 2007; Veazey et al., 2009; Modugno et al., 2010; Pal et al., 2010; Smania et al., 2010; Sproesser et al., 2010; Dobkin et al., 2011, 2021; Edwards et al., 2013; Naismith et al., 2013; Okai et al., 2013; Rios Romenets et al., 2013, 2015; Shirota et al., 2013; Peña et al., 2014; Petrelli et al., 2014; Troeung et al., 2014; Calleo et al., 2015; Dashtipour et al., 2015; Lee et al., 2015, 2018; Bega et al., 2016; Brys et al., 2016; Patel et al., 2016; Picelli et al., 2016; Fan et al., 2017; Ghielen et al., 2017; Tröster et al., 2017; Videnovic et al., 2017; Xu and Xia, 2017; Yu et al., 2017; Cheung et al., 2018; Cohen et al., 2018; Kong et al., 2018; Michels et al., 2018; Pérez-de la Cruz, 2018; Tollár et al., 2018; Willis et al., 2018; Kwok et al., 2019; Rodgers et al., 2019; Rutten et al., 2019; Sacheli et al., 2019; Solla et al., 2019; Wuthrich and Rapee, 2019; Fellman et al., 2020; Kalbe et al., 2020; Kraepelien et al., 2020; Li et al., 2020; Moon et al., 2020; Wu et al., 2020, 2021; You and She, 2020; Zheng et al., 2020; Zhuang et al., 2020; Aftanas et al., 2021; Han et al., 2021; Schmidt et al., 2021; Bogosian et al., 2022) and 2 RCTs (Stallibrass et al., 2002; Wu et al., 2019) were multi-arm trials.

**Table 1:** Characteristics of included trails in this network meta-analysis.

**References (country)**	**Sample (I/C) (Mean age)**	**Duration of disease, years**	**Hoehn-Yahr**	**Gender (M/F)**	**Intervention**	**Details of interventions**	**Measured outcomes**
Aftanas et al. (2021) (Russia)	23/23 (63.3)	I: 7.0 ± 4.0 C: 5.6 ± 4.0	NA	21/25	I:10-Hz rhythmic TMS C: Placebo	M1: 100% MT, 4,000 pulses/day; lDLPFC: 110% MT, 3,000 pulses/ day; 20 consecutive days	HDRS
Bega et al. (2016) (America)	7/7 (67.3)	NA	I: 2.3 ± 0.4 C: 2.4 ± 0.5	11/3	I: Yoga C: Resistance exercise	60 min each, twice/week, 12 weeks	BDI
Bogosian et al. (2022) (Britain)	30/30 (60.9)	I: 5.22 ± 3.55 C: 6.43 ± 3.85	NA	30/30	I: Mindfulne- ss interven- tion C: waitlist	60min each, once /week 8 weeks	HDRS
Brys et al. (2016) (America)	20/15 (64.5)	I: 7.30 ± 5.60 C: 4.50 ± 2.20	NA	22/13	I:10-Hz rTMS C: Placebo	Bilateral M1: 2000 (1,000 each side) pulses/day; lDLPFC: 2,000 pulses/ day; 10 consecutive days	HDRS
Calleo et al. (2015) (America)	7/4 (62.9)	NA	NA	NA	I: CBT C: TAU	30–40 min each, 8 sessions, 12 weeks	HDRS
Cohen et al. (2018) (Israel)	21/21 (65.6)	I: 4.70 ± 3.40 C: 5.60 ± 3.70	I: 2.0 (2.0–2.5) C: 2.0 (2.0–2.5) [Median (range)]	32/10	I: 1-Hz rTMS (M1) 10-Hz (PFC) C: Placebo	M1: 110% MT, 900 pulses/ day; Bilateral PFC: 100 MT, 800 pulses/ day; 90 consecutive days	BDI
Cheung et al. (2018) (Australia)	10/10 (64.7)	NA	I: 1.8 ± 1.0 C: 1.3 ± 0.5	NA	I: Yoga C: Waitlist	60 min each, twice/week, 12 weeks	BDI
Craig et al. (2006) (America)	18/14 (63.3)	NA	I: 1.8 ± 1.0 C: 1.3 ± 0.5	23/9	I: Massage therapy C: Music therapy	45 min each, twice/week, 4 weeks	BDI
Dashtipour et al. (2015) (America)	6/5 (63.3)	I: 2.9 ± 1.5 C: 4.5 ± 3.3	I: 1.8 ± 0.5 C: 1.3 ± 0.5	NA	I: LSVT BIG therapy C: Aerobic exercise	60min each, 4 times/week, 4 weeks	BDI
Dobkin et al. (2011) (America)	41/39 (64.6)	I: 6.53 ± 5.53 C: 6.13 ± 5.56	NA	48/32	I: CBT C: Clinical monitoring	60–75 min each, once/ week, 10 weeks	BDI
Dobkin et al. (2021) (America)	45/45 (66.8)	I: 5.4 ± 5.01 C: 5.24 ± 5.13	NA	90/0	I: CBT C: TAU	60 min each, once/week 10 weeks	HDRS
Edwards et al. (2013) (America)	44/43 (68.8)	I: 7.25 ± 6.14 C: 6.63 ± 4.89	I: 2 (1–3) C: 2 (1–3) [Median (range)]	54/33	I: SPOT C: Waitlist	3 months	CES-D
Fan et al. (2017) (China)	18/18 (64.1)	NA	NA	15/21	I: Qigong C: TAU	60 min each, 5 times/week, 8 weeks	POMS-D
Fellman et al. (2020) (Finland)	26/26 (65.2)	I: 5.2 ± 3.2 C: 6.0 ± 6.1	NA	18/34	I: Working memory train- ing C: Active quiz task	45 min each, 3 times/week, 7 weeks	GDS-30
Ghielen et al. (2017) (Netherlands)	19/19 (63.1)	I: 10.5 ± 5.7 C: 12.3 ± 4.3	NA	7/31	I: Body awar- eness training C: AU	60 min each, twice/week, 6 weeks	BDI
Han et al. (2021) (China)	50/50 (68.2)	I: 36 (24–120) C: 36 (24-111) [Median (range)] (months)	I: 2.56 ± 0.78 C: 2.80 ± 1.04	49/51	I: VR + Wuqinxi C: TAU	VR: 20–30 min each, 3 times/week, 2 weeks Wuqinxi: 40 min each, 3 times/week, 6 weeks	HDRS
Kalbe et al. (2020) (Germany)	33/31 (67.6)	G1: 13 (2-20) G2: 12 (9-20)	NA	40/24	I: Multidoma- in group cognitive training C: Streching exercise	90 min each, twice/week, 6 weeks	BDI-II
Kong et al. (2018) (Singapore)	20/20 (64.6)	I: 68.8 ± 45.5 C: 87.2 ± 53.2 (months)	NA	13/27	I: Acupunctu- re C: Placebo	70 min each, twice/week, 5 weeks	GDS
Kraepelien et al. (2020) (Sweden)	38/39 (66.0)	I: 8.3 ± 4.4 C: 9.6 ± 5.7	NA	30/47	I: ICBT C: Waiting list	10 weeks	HADS
Kwok et al. (2019) (China)	71/67 (63.6)	NA	NA	65/73	I: Yoga C: Streching exercise	90 min each, once/week, 8 weeks	HDRS
Lee et al. (2015) (Korea)	10/10 (69.3)	NA	NA	10/10	I: VR C: Balance training	Dance exerci- se using Nintendo Wii TM fit game, 45 min each, 5 times/week 6 weeks	BDI
Lee et al. (2018) (Korea)	25/16 (65.8)	I: 4.5 ± 3.3 C: 4.4 ± 3.0	NA	17/24	I: Qi dance C: waitlist	60 min each, twice/week, 8 weeks	BDI
Li et al. (2020) (China)	24/24 (61.6)	I: 5.48 ± 3.69 C: 6.46 ± 5.17	I: 1.85 ± 0.63 C: 1.83 ± 0.64	16/32	I: rTMS C: Placebo	Bilateral M1: 80% RMT, 2,000 pulses/ day, 5 consecutive days	HDRS
Michels et al. (2018) (America)	9/4 (69.2)	NA	I: 2.11 ± 0.33 C: 2.50 ± 1.00	NA	I: Dance C: Health ed- ucation	60 min each, twice/week, 10 weeks	BDI
Modugno et al. (2010) (Italy)	10/10 (62.6)	I: 3.0 ± 0.22 C: 3.5 ± 0.17	I: 10 ± 1.8 C: 9.4 ± 1.1	10/10	I: Active theater C: Physiothe- rapy	6 h each, 18 h/month, 3 years	HDRS
Moon et al. (2020) (America)	8/9 (66.1)	I: 4.25 ± 2.1 C: 5.33 ± 3.3	I: 2 (2–2) C: 2 (2–2)	10/7	I: Qigong C: Placebo	15–20 min each, twice/day, 12 weeks	GDS
Naismith et al. (2013) (Britain)	35/15 (67.4)	I: 6.0 ± 5.5 C: 8.1 ± 5.6	I: 2.1 ± 0.5 C: 2.1 ± 0.5	36/14	I: Cognitive training C: Waitlist	120 min each, twice/day, 7 weeks	BDI-II
Okai et al. (2013) (Britain)	28/17 (58.8)	I: 10.5 ± 6.0 C: 8.8 ± 5.6	NA	31/14	I: CBT C: waitlist	12 sessions, 12 weeks	BDI
Pal et al. (2010) (Hungary)	12/10 (68.5)	I: 6 (3-9.5) C: 6.5 (3.75–10.5) [Median (range)]	NA	11/11	I: 5-Hz rTMS C: Placebo	lDLPFC: 90% RMT, 600 pulses/day; 10 consecutive days	BDI
Patel et al. (2016) (America)	14/14 (63.9)	NA	NA	16/12	I: CBT C: sleep hygi- ene advice	6 weeks	PHQ-9
Paus et al. (2007) (Germany)	18/18 (63.5)	I: 7.4 ± 4.3 C: 7.9 ± 4.7	I: 2.7 ± 0.6 C: 2.5 ± 0.4	23/13	I: BLT C: Placebo	7,500 lux for 30 min daily for 14 days	BDI
Peña et al. (2014) (Spain)	22/22 (67.8)	I: 5.6 ± 4.6 C: 7.4 ± 5.7	NA	27/17	I: Cognitive training C: Occupatio- nal group activities	60 min each, 3 times/week, 12 weeks	GDS
Pérez-de la Cruz (2018) (Spain)	15/15 (65.1)	I: 7.1 ± 2.042 C: 7.7 ± 3.025	I: 2.81 ± 0.22 C: 2.76 ± 1.02	15/15	I: Aquatic Ai chi C: Stretching exercise	45 min each, twice/week, 10 weeks	GDS
Petrelli et al. (2014) (Germany)	22/21 (69.0)	I: 66.2 ± 39.5 C: 65.0 ± 52.8 (months)	NA	22/21	I: NEUROvit- alis C: Waiting list	90 min each, twice/week, 6 weeks	BDI-II
Picelli et al. (2016) (Italy)	9/8 (71.4)	I: 11.2 ± 5.6 C: 10.8 ± 4.1	NA	9/8	I: Treadmill training C: Regular social interactions	45 min each, 3 times/week, 4 weeks	BDI
Rodgers et al. (2019) (Australia)	18/18 (63.7)	NA	NA	16/20	I: Mindfulne- ss interven- tion C: waitlist	120 min each, 6 sessions, 8 weeks	GDS-15
Rios Romenets et al. (2013) (Canada)	6/6 (67)	I: 5.2 ± 1.8 C: 5.2 ± 4.4	NA	11/1	I: CBT+BLT C: Placebo	CBT: 90 min each, once/week, 6 weeks BLT: 10,000 lux for 30 min daily	BDI
Rios Romenets et al., 2013 (Canada)	18/15 (63.7)	I: 7.7 ± 4.6 C: 5.5 ± 4.4	NA	19/14	I: Dance C: waitlist	60 min each, twice/week, 12 weeks	BDI
Rutten et al. (2019) (Netherlands)	35/37 (62.5)	NA	I: 2.1 ± 0.6 C: 2.4 ± 0.7	40/32	I: BLT C: Placebo	10,000 lux for 30 min twice/day for 3 months	HDRS
Sacheli et al. (2019) (Britain)	20/15 (67.2)	I: 3.91 ± 2.85 C: 5.17 ± 4.26	NA	22/13	I: Aerobic exercise C: Stretching exercise	40–60 min each, 3 times/week, 12 weeks	BDI
Schmidt et al. (2021) (Germany)	28/26 (67.3)	I: 13.34 ± 3.84 C: 13.96 ± 3.33	NA	36/18	I: NEUROvit- alis C: Stretching exercise	90 min each, twice/week, 6 weeks	BDI-II
Smania et al. (2010) (Italy)	28/27 (67.5)	I: 10.39 ± 4.76 C: 8.63 ± 5.39	I: 3.0 ± 0.1 C: 3.1 ± 0.3	29/26	I: Balance training C: Stretching exercise	50 min each, 3 times/week, 7 weeks	GDS
Shirota et al. (2013) (Japan)	34/36 (66.8)	I: 7.8 ± 6.6 C: 7.6 ± 4.4	NA	31/39	I:10-Hz rTMS C: Placebo	SMA: 110% RMT, 1,000 pulses/day, 8 weeks	HDRS
Stallibrass et al. (2002) (Britain)	29/29/30 (65.0)	G1: 4.8 ± 4.3 G2: 4.7 ± 3.7 G3: 4.9 ± 3.5	NA	61/27	G1: Alexand- er technique G2: Massage G3: TAU	40 min each, twice/week, 12 weeks	BDI
Solla et al. (2019) (Italy)	10/10 (67.5)	I: 4.4 ± 4.5 C: 5.0 ± 2.9	I: 2.1 ± 0.6 C: 2.3 ± 0.4	13/7	I: Dance C: TAU	90 min each, twice/week, 12 weeks	BDI-II
Sproesser et al. (2010) (Brazil)	8/8 (59.0)	I: 9.0 ± 4.0 C: 7.0 ± 4.0	I: 2.4 ± 0.2 C: 2.2 ± 0.4	9/7	I: Psychother- apeutic intervention, C: Waiting list	90 min each, twice/month, 12 months	BDI
Tollár et al. (2018) (Netherlands)	35/20 (67.4)	I: 6.7 ± 2.3 C: 7.1 ± 2.8	NA	29/26	I: VR C: TAU	The Xbox virtual reality exergame: 60 min each, 15 sessions over 3 weeks	BDI
Troeung et al. (2014) (Australia)	11/7 (66.0)	I: 5.7 ± 5.5 C: 4.29 ± 3.25	NA	12/6	I: CBT C: waitlist	120 min each, once/week, 8 weeks	DASS-D
Tröster et al. (2017) (America)	131/35 (60.3)	I: 12.1 ± 4.9 C: 11.7 ± 4.1	NA	84/52	I: DBS C: waitlist	Receive stim-ulation imme- diately (7 days) after device impla- ntation was completed	HDRS
Veazey et al. (2009) (America)	4/4 (71.0)	NA	NA	8/0	I: Telephone CBT C: Phone calls about the patients' general wellbeing	Average 41 min each, once/week, 8 weeks	PHQ-9
Videnovic et al. (2017) (America)	16/15 (63.1)	I: 5.94 ± 3.57 C: 8.38 ± 3.71	I: 2.1 ± 0.3 C: 2.3 ± 0.5	13/18	I: BLT C: Placebo	10,000 lux for 60 min twice/ day for 14 days	BDI
Wade et al. (2003) (Britain)	53/41 (70.9)	NA	NA	56/38	I: Multidisci- plenary reha- bilitation C: Waiting list	120 min each, once/week, 24 weeks	HDRS
Willis et al. (2018) (Australia)	10/10 (68.9)	NA	NA	17/13	I: Polychrom- atic light C: Placebo	3,000 lux for 1 h for 2 weeks	BDI-II
Wu et al. (2019) (China)	50/50/50 (60.1)	G1: 5.8 ± 1.6 G2: 5.5 ± 1.4 G3: 6.0 ± 1.7	NA	89/61	G1:1-Hz/5-Hz rTMS G2: Tradition- al rehabilita- tion G3: G1+G2	lDLPFC: 80% MT, 1,600 pulses/day; 20 days in 4 weeks	HDRS
Wu et al. (2020) (China)	28/26	I: 5.8 ± 2.6 C: 5.7 ± 3.5	I: 2.4 ± 0.8 C: 2.5 ± 3.6	30/24	I: tDCS C: Placebo	DLPFC: (F3, F4): 1.2 mA, 20 min, 24.75 cm^2^, 5 times/week, 4 weeks	HDRS
Wu et al. (2021) (China)	49/49 (65.1)	I: 4.97 ± 3.91 C: 5.66 ± 3.81	NA	56/42	I: Combined exercise C: TAU	50 min each, 3 times/week, 8 weeks	GDS
Wuthrich and Rapee (2019) (Australia)	6/5 NA	NA	NA	NA	I: Telephone CBT C: waitlist	10 weeks	GDS
Xu and Xia (2017) (China)	35/35 (72.5)	I: 7.3± 2.2 C: 7.1 ± 2.1	NA	42/28	I: Auricular pressure + pointed psychological nursing C: TAU	3–4 times/day, 1–3 min/time, 9 days	SDS
You and She (2020) (China)	35/35 (68.7)	I: 4.17 ± 0.35 C: 4.21 ± 0.24	NA	37/33	I: Tai chi C: Stretching exercise	60 min each, twice/week, 24 weeks	HDRS
Yu et al. (2017) (China)	31/33 (67.6)	I: 2.76 ± 1.56 C: 2.64 ± 1.49	NA	30/34	I: 5-Hz rTMS C: Placebo	Bilateral DLPFC: 80% MEP, 1,600 pulses/ day, consecutive 10 days	HDRS
Zheng et al. (2020) (China)	35/35 (72.9)	I: 6.34 ± 0.27 C: 6.39 ± 0.25	NA	41/29	I: Resistance exercise C: TAU	50 min each, 3 times/week, 4 weeks	HDRS
Zhuang et al. (2020) (China)	19/14 (61.0)	I: 70.37 ± 52.26 C: 68.57± 45.29 (month)	I: 2 (1.5–2.5) C: 2.25 (1.75–3.0) [Median (range)]	15/18	I: 1-Hz rTMS C: Placebo	rDLPFC: 110% RMT, 1,200 pulses/ day, consecu- tive 10 days	HDRS

I, intervention; C; control; M, male; F, female; BLT, bright light therapy; CBT, cognitive behavioral therapy; DBS, deep brain stimulation; TAU, treatment as usual; TMS, transcranial magnetic stimulation; rTMS, repetitive transcranial magnetic stimulation; tDCS, transcranial direct current stimulation; VR, virtual reality; LSVT-BIG, Lee Silverman Voice Therapy BIG; lDLPFC or rDLPFC, left or right dorsolateral prefrontal cortex; MT, motor threshold; RMT, resting motor threshold; M1, primary motor cortex; SMA, supplementary motor area; MEP, motor evoked potential; BDI, Beck depression inventory; HDRS, Hamilton Depression Rating Scale; GDS, Geriatric Depression Scale; DASS-D, Depression, Anxiety, Stress Scale-depression; PHQ, Patient Health Questionnaire; SDS, Self-Rating Depression Scale.

### Quality assessment

**Figure 2:**
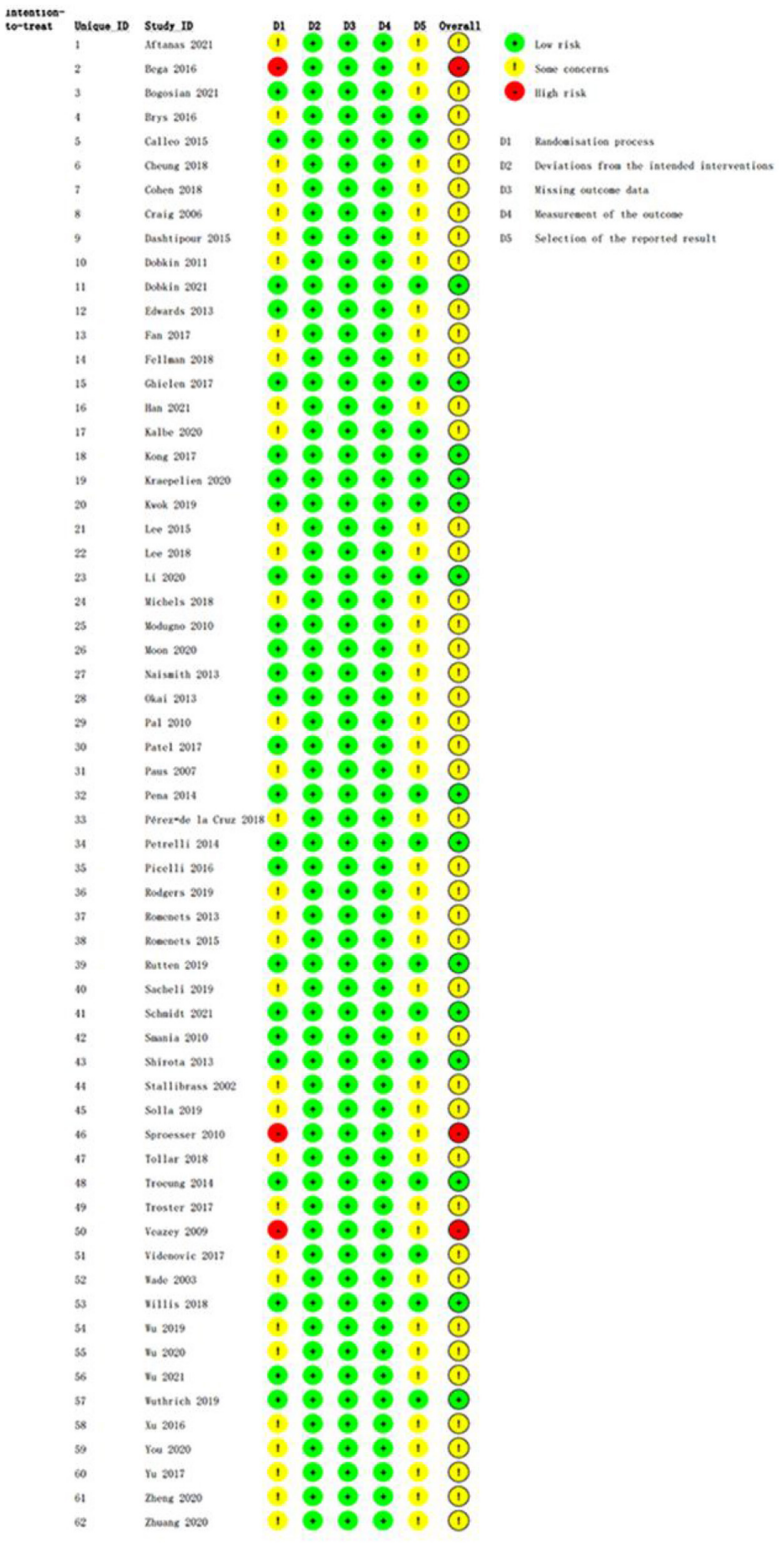
Quality assessment of the eligible studies.

The methodological quality assessments of the eligible RCTs are shown in Figure 2, ranging from low to high risk. All eligible RCTs met the criteria for deviation from the intended interventions, missing outcome data, and outcome measurements. Three trials had obvious flaws in the domain of randomization; thus, their risk of bias was rated as high. Forty-five RCTs were rated as “some concerns” due to weaknesses in the randomization process or selection of the reported result domains.

### Network meta-analysis

**Figure 3:**
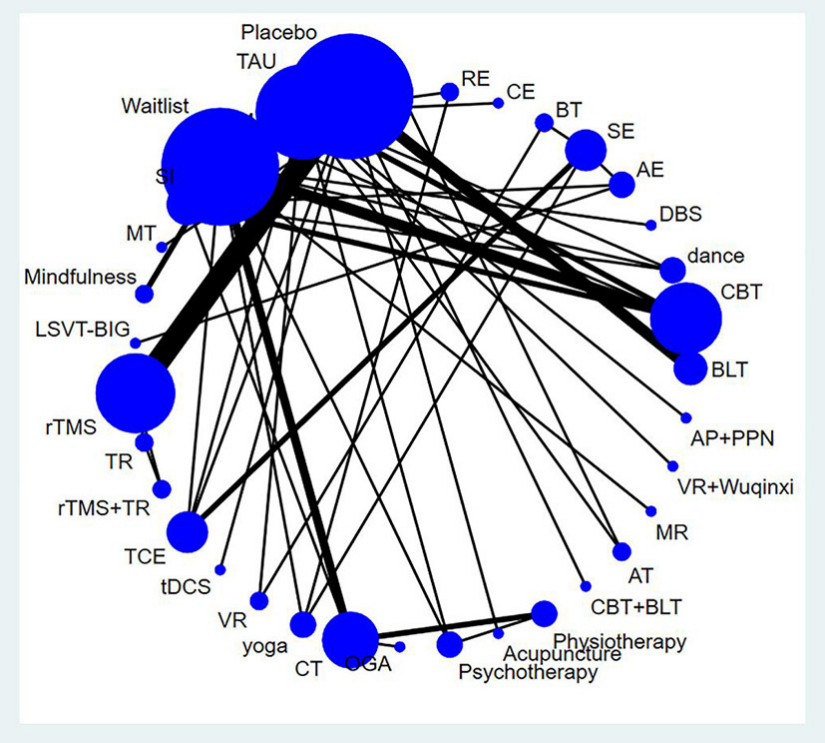
Network map for depression. AE, Aerobic exercise; AP+PPN, Auricular pressure and pointed psychological nursing; AT, Alexander technique; BLT, Bright light therapy; BT, Balance training; CBT, Cognitive behavior therapy; CBT + BLT, Cognitive behavioral therapy and bright light therapy; CE, Combined exercise; CM, Clinical mo- nitoring; CT, Cognitive training; DBS, Deep brain stimulation; MR, Multidisciplenary rehabilitation; MT, music therapy; OGA, Occupational group activities; RE, resistance exercise; SE, Stretching exerc-ise; SI, Supportive instruction; TAU, Treatment as usual; TCE, Traditional Chinese exercise; TMS, Transcranial magnetic stimulation; TR, Traditional rehabilitation; VR, Virtual reality; rTMS, repetitive tra- nscranial magnetic stimulation; tDCS, Transcranial direct current stimulation.

Figure 3 shows the network map of the different non-pharmacological interventions for depression and indicates that comparisons among CBT, TMS, BLT, and CT were common. An NMA was conducted to compare the effects of different interventions on depression in PD patients. Supplementary Table S1 shows the relative effects of the different interventions on depression. The league table shows the pairwise comparisons of 35 non-pharmacological interventions for depression in PD subjects. Compared to occupational group activities, dance (SMD: −3.23; 95% CI: −6.05–−0.41), LSVT-BIG therapy (SMD: −3.36; 95% CI: −6.27–−0.45), CBT (SMD: −2.76; 95% CI: −5.31–−0.21), aerobic exercise (SMD: −2.69; 95% CI: −5.07–−0.30), mindfulness intervention (SMD: −2.31; 95% CI: −4.06–−0.56), TCE (SMD: −2.14; 95% CI: −4.10–−0.18), DBS (SMD: −2.17; 95% CI: −4.07–−0.27), CT (SMD: −1.85; 95% CI: −3.16–−0.53), waitlist (SMD: −1.76; 95% CI: −3.26–−0.26), and physiotherapy (SMD: −1.72; 95% CI: −3.29–−0.14) all showed significant improvement effects on depression. Compared to stretching exercises, dance (SMD: −2.67; 95% CI: −4.91–−0.44), CBT (SMD: −2.2; 95% CI: −4.09–−0.31), and TCE (SMD: −1.58; 95% CI: −2.52–−0.63) showed significant improvement effects on depression. Compared to TAU, dance (SMD: −1.70; 95% CI: −3.25–−0.14) and CBT (SMD: −1.23; 95% CI: −2.22–−0.23) had significantly positive effects.

### Rank probability

**Figure 4:**
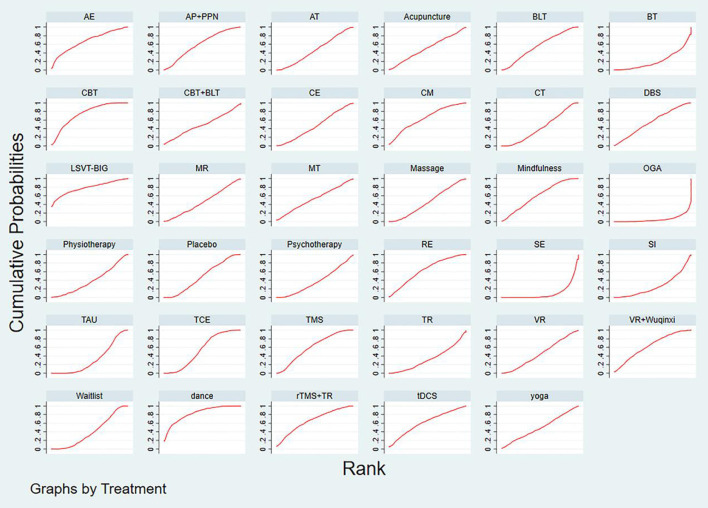
The SUCRA plot based on cumulative probabilities of interventions. AE, Aerobic exercise; AP+PPN, Auricular pressure and pointed psychological nursing; AT, Alexander technique; BLT, Bright light therapy; BT, Balance training; CBT, Cognitive behavior therapy; CBT + BLT, Cognitive behavioral therapy and bright light therapy; CE, Combined exercise; CM, Clinical mo- nitoring; CT, Cognitive training; DBS, Deep brain stimulation; MR, Multidisciplenary rehabilitation; MT, music therapy; OGA, Occupational group activities; RE, resistance exercise; SE, Stretching exerc-ise; SI, Supportive instruction; TAU, Treatment as usual; TCE, Traditional Chinese exercise; TMS, Transcranial magnetic stimulation; TR, Traditional rehabilitation; VR, Virtual reality; rTMS, repetitive tra- nscranial magnetic stimulation; tDCS, Transcranial direct current stimulation.

The SUCRA plot and values are shown in Figure 4 and Table 2, respectively. The SUCRA values and the plot revealed that the treatments' comparative efficacy in improving depression was, in order: dance >LSVT-BIG>CBT>rTMS + traditional rehabilitation>aerobic exercise>clinical monitoring>VR + Wuqinxi>tDCS>resistance exercise>mindfullness>auricular pressure and pointed psychological nursing>TMS>TCE>music therapy>acupuncture>DBS>BLT>yoga>multidisciplinary rehabilitation>massage>cognitive training>placebo>the alexander technique>CBT and BLT>combined exercise>VR>waitlist>physiotherapy>psychotherapy>traditional rehabilitation>TAU>supportive instruction>balance training>stretching exercise>occupational group activities.

**Table 2:** SUCRA values of 35 non-pharmacological interventions.

**Treatments**	**SUCRA**
BLT	54.1
CBT	73.6
Dance	**82.3**
DBS	55.5
Aerobic exercise	68.5
Stretching exercise	9.1
Balance training	21.3
Combined exercise	44.9
Resistance exercise	61.3
Massage	47.8
Placebo	46.4
TAU	31
Clinical monitoring	62.9
Waitlist	42.3
Supportive instruction	25.5
Music therapy	57.1
Mindfulness	60.9
LSVT-BIG therapy	77.4
TMS	57.4
Traditional rehabilitation	31.7
TMS and traditional rehabilitation	68.6
Traditional Chinese exercise	57.3
tDCS	62
VR	42.4
yoga	52.8
Cognitive training	46.7
Occupational group activities	7.4
Psychotherapy	37
Acupuncture	56.5
Physiotherapy	40.7
CBT and BLT	45.5
Alexander technique	46.2
Multidisciplenary rehabilitation	52.8
VR and Wuqinxi	62.8
Auricular pressure and pointed psychological nursing	60.4

BLT, bright light therapy; CBT, cognitive behavioral therapy; DBS, deep brain stimulation; TAU, treatment as usual; TMS, transcranial magnetic stimulation; rTMS, repetitive transcranial magnetic stimulation; tDCS, transcranial direct current stimulation; VR, virtual reality; LSVT-BIG, Lee Silverman Voice Therapy BIG. The bold value means the best SUCRA value.

### Consistency analysis

**Figure 5:**
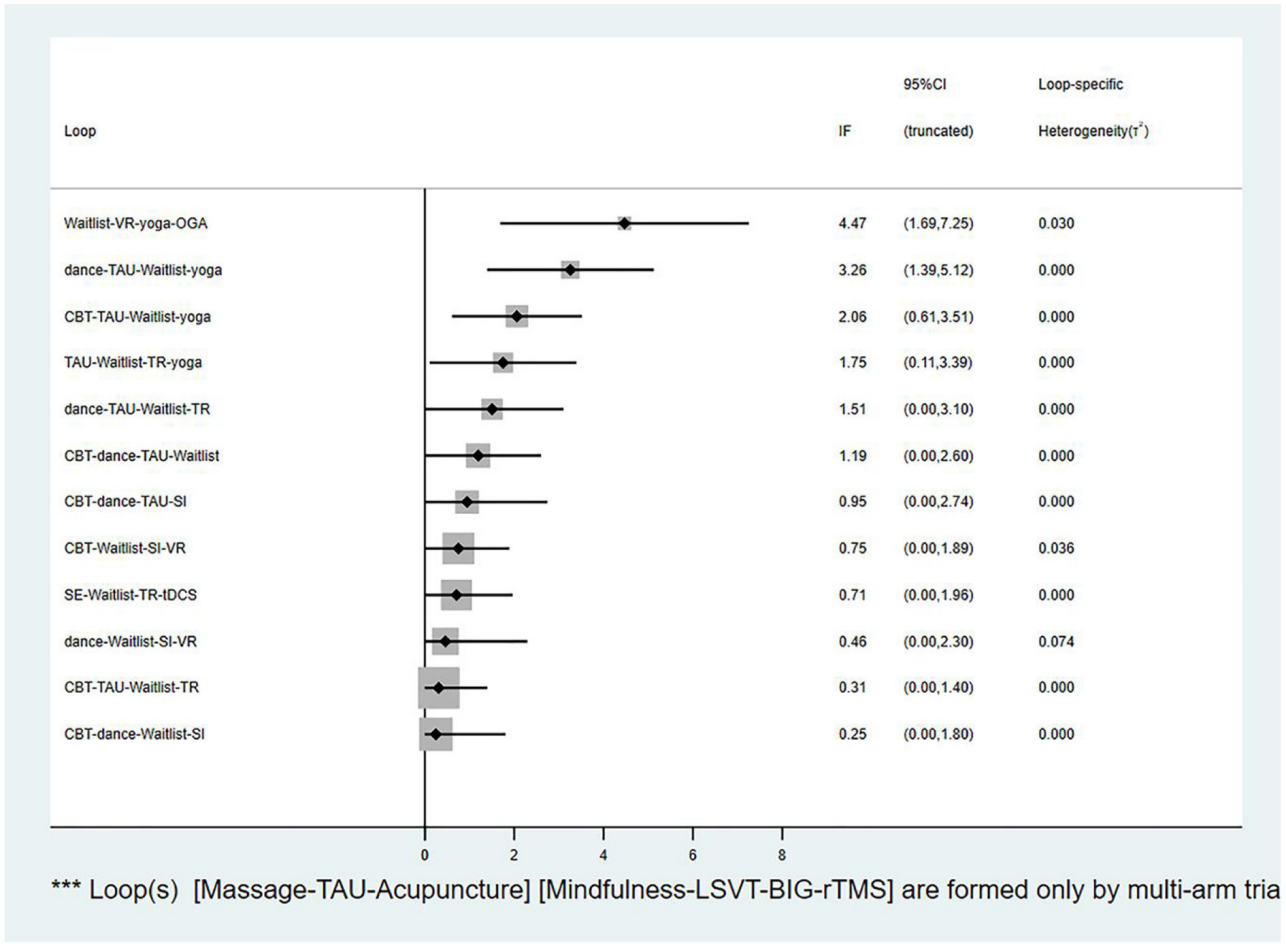
The loop inconsistency plot. VR, Virtual reality; OGA, Occupational group activities; TAU, Treatment as usual; CBT, Cognitive behavior therapy; TR, Traditional rehabilitation; SI, Supportive instruction; SE, Stretching exercise; tDCS, Transcranial direct current stimulation.

The global inconsistency analysis of this NMA showed a *P*-value of 0.0038, indicating significant inconsistency. Moreover, the results of the node-splitting analysis indicated that the four indirect comparisons had inconsistencies (*P* < 0.05). The results are summarized in Supplementary Table S2. Therefore, we used an inconsistency model to perform the NMA. Considering that two triangular loops were formed by the multi-arm trials, I^2^ quadratic loop consistencies were examined if they were inconsistent. The 95% CI of the four closed loops included 0, indicating significant inconsistency (Figure 5). However, the lower CI was close to 0, which indicated low consistency. Although the NMA revealed an obvious overall inconsistency, the comparisons of inconsistency using node-splitting analysis and the loop-specific method both included a small number of studies and sample sizes. Therefore, the inconsistencies were not significant. Inconsistency tests can be affected by limited numbers of studies and small sample sizes, making accurate evaluation difficult. Due to the low heterogeneity of the data (τ < *0.5*), a meta-regression analysis of potential effects was not performed (Turner et al., 2012).

### Publication analysis

**Figure 6:**
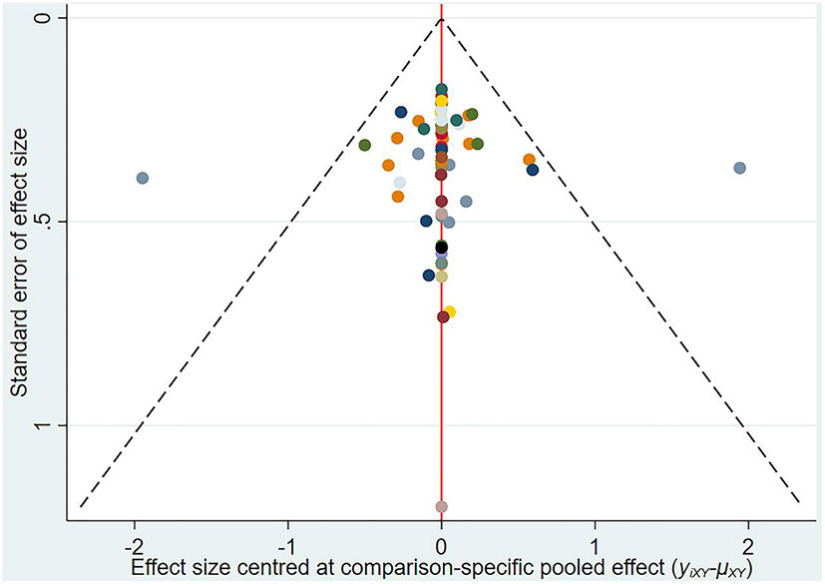
The funnel plot of depression.

A comparison-adjusted network funnel plot with a random model was constructed for the outcome (Figure 6). The funnel plot was symmetric, demonstrating that there was no significant risk of publication bias in our study.

## Discussion

This NMA was conducted to synthesize existing evidence from 62 RCTs involving 35 non-pharmacological interventions for depressive symptoms using a comprehensive search. Direct and indirect comparisons were conducted to analyze the efficacy of different non-pharmacological interventions on depressive symptoms. The SUCRA values revealed that the best non-pharmacological intervention was dance, followed by LSVT-BIG therapy and CBT. Despite the lack of strong evidence, current guidelines recommend ECT, physical exercise, and psychotherapy to treat depression in PD patients (Olanow et al., 2001; Goodarzi et al., 2018). However, our NMA did not include RCTs on the effect of ECT on depression.

Although two conventional meta-analyses showed that dance had no significant effect on depression (Zhang et al., 2019; Wang et al., 2022), most original trials indicated a supportive effect of dance on depression compared to no intervention or TAU. In addition, our study demonstrated that dance was the best non-pharmacological intervention based on its high SUCRA value (82.3%). Dance is a multicomponent activity that involves moving one's muscles, maintaining one's balance, maintaining auditory, visual, and sensory reactions, memory, perception, expression, and social interactions (Kattenstroth et al., 2010). Dance can create a sense of pleasure by combining physical exercise and mental regulation, which contributes to increased compliance and continued attendance (Earhart, 2009; Hackney and Earhart, 2010; Goodarzi et al., 2018). The sense of pleasure may evoke positive emotions by stimulating basal ganglia loops and reward systems (Hackney and Earhart, 2009). Meanwhile, music used in dance can increase the release of dopamine from the ventral striatum and ventral tegmental area to alleviate depressive symptoms (Weintraub et al., 2005). However, this ranking has an underlying uncertainty and may not fully reflect reality.

LSVT-BIG therapy is a type of exercise model that focuses on training of amplitude together with sustained attention and perception of a single movement during exercise, thus enabling patients to participate physically and mentally in functional activities with great sustainability (Fox et al., 2012). Our study indicated that LSVT-BIG therapy is the second-ranked non-pharmacological intervention according to its SUCRA value (77.4%), whereas only one RCT showed a positive effect of LSVT-BIG therapy on depression compared with general exercise (Dashtipour et al., 2015). However, the small sample sizes of the studies limited the accuracy and generalization of the conclusions; therefore, more large-scale and strictly designed trials are needed. The mechanism by which LSVT-BIG therapy is effective in reducing depressive symptoms remains unclear. Additionally, current trials lack focus on the long-term effects of LSVT-BIG therapy. Thus, the maintenance of long-term effects and exploration of the underlying mechanisms should be given more attention in future studies.

CBT, as a psychotherapy, is the third-ranked non-pharmacological intervention according to its SUCRA value (73.6%), which is similar to the recommendations of the guidelines and results of conventional meta-analyses (Goodarzi et al., 2018; Zhang et al., 2020; Hong et al., 2021). CBT is a problem-oriented approach that aims to enhance a patient's coping skills. This approach involves both therapists and patients and aims to help patients overcome negative moods, dysfunctional thoughts, and behaviors by modifying their way of thinking and behaving (Farley and Koshland, 2005; Schrag et al., 2007; O'cleirigh et al., 2019; Sahranavard et al., 2019). Among the conventional meta-analyses that demonstrated that CBT had a significant positive effect on the improvement of depression (Troeung et al., 2013; Xie et al., 2015; Zhang et al., 2020; Hong et al., 2021), a meta-analysis showed that CBT had a larger effect size than antidepressant treatments (Troeung et al., 2013). To date, most trials have indicated that CBT plays a beneficial role in depression disorders in patients with PD. One review suggested that CBT is more appropriate for patients with PD without dementia, as patients with dementia are usually excluded from trials (Egan et al., 2015). In short, CBT can be considered an important approach for first-line or adjunctive treatment of depression in PD.

Interestingly, our results indicate that most non-pharmacological interventions examined in our study had no significant effect on alleviating depressive disorders. This may be due to the use of antidepressant medications and the progression of PD (Schrag et al., 2001; Bhattacharjee et al., 2018; Demarco et al., 2021). Moreover, depression in PD patients is associated with cognitive impairment. As the disease progresses, cognitive deficits negatively affect depressive symptoms (Van Uem et al., 2018). Although most included trials in our study excluded PD patients with dementia, the impact of mild cognitive impairment cannot be ignored. In addition, it is difficult for short-term non-pharmacological interventions to improve neuroanatomical degeneration (Mcdonald et al., 2003). According to their SUCRA values, some non-pharmacological interventions were ranked below placebo and waitlist. This may be due to a lack of sufficient evidence showing the efficacy of these interventions for depression in PD. Although a global inconsistency was present in our NMA, the local inconsistency was found to be weak in the node-split test, and inconsistencies were mainly due to the triangular loops from two multi-arm trials. Therefore, related results should be interpreted with caution.

Conventional meta-analyses, including RCTs, showed that yoga, BLT, rTMS, and psychotherapy significantly improved depression (Ban et al., 2021; Chen et al., 2021; Hong et al., 2021; Huang et al., 2021); however, the evidence was relatively weak. Although a previous systematic review showed that physical exercise is beneficial for depression, it did not provide a quantitative analysis of the intervention effects (Cusso et al., 2016). Guidelines and reviews suggest that ECT may be an effective therapy for depression (Bhattacharjee et al., 2018; Goodarzi et al., 2018); however, the current systematic review with meta-analysis did not identify any relevant RCTs of ECT (Takamiya et al., 2021). Therefore, our study did not compare ECT with the other non-pharmacological interventions. Recently, a systematic review and meta-analysis by Xie et al. revealed that psychodynamic psychotherapy was superior to CBT (Xie et al., 2015). According to the eligibility criteria of our study, we did not include RCTs on the effect of psychodynamic psychotherapy on depression in patients with PD. Psychotherapy in combination with non-CBT requires further high-quality evidence to explore its efficacy for treating depression in PD patients.

This NMA has several limitations. First, only RCTs that focused on PD patients with mean Hoehn–Yahr stage values of 1–3 were included in our NMA; thus, the results of this NMA may not be generalizable to all PD patients. Second, there was heterogeneity in the frequencies, durations, and periods of the non-pharmacological interventions. Third, although we comprehensively searched for non-pharmacological interventions for depression in patients with PD, the language was restricted to Chinese and English, which may have contributed to selection bias. Fourth, our NMA had obvious global inconsistencies, which may be related to the small sample size, short-term effects, discrepancies in eligibility criteria, different baseline characteristics, various measurement tools, different severities of depression, and different severities of PD. Thus, the accuracy and generalization of the conclusions are limited. Fifth, many comparisons of the interventions included only a small number of trials, which may have affected the accuracy of the conclusions. Lastly, most studies did not report concealed allocation, which may have led to selection and performance biases. Therefore, strictly designed RCTs with larger sample sizes are needed in the future.

## Conclusion

To the best of our knowledge, this is the first NMA to comprehensively summarize the existing RCTs of 35 different non-pharmacological interventions used for depressive symptoms. The results showed non-significant effects of most non-pharmacological interventions on depression. According to its SUCRA values, dance may be the preferred non-pharmacological intervention for improving depression, followed by LSVT-BIG therapy and CBT. Consequently, a larger sample size and stronger high-quality trials are required to draw more reliable results regarding the efficacy of non-pharmacological interventions for depression in subjects with PD. The results of this study could provide evidence and a reference to healthcare providers and clinicians when choosing effective interventions to improve the quality of life and health status of patients with PD.

## Data availability statement

The original contributions presented in the study are included in the article/Supplementary material, further inquiries can be directed to the corresponding author.

## Author contributions

YW, XS, and YJ conceived and designed the study. YW and XS searched the literature and contributed to writing of original manuscript, data acquisition and analysis, and responsible for the software. FL, QL, and YJ were responsible for revising and reviewing. All authors contributed to the article and approved the submitted version.

## Conflict of interest

The authors declare that the research was conducted in the absence of any commercial or financial relationships that could be construed as a potential conflict of interest.

## Publisher's note

All claims expressed in this article are solely those of the authors and do not necessarily represent those of their affiliated organizations, or those of the publisher, the editors and the reviewers. Any product that may be evaluated in this article, or claim that may be made by its manufacturer, is not guaranteed or endorsed by the publisher.
