# Supplementary material for: Efficacy of non-pharmacological interventions for depression in individuals with Parkinson's disease: A systematic review and network meta-analysis
Source: Front Aging Neurosci. 2022 Nov 10;14:1050715. doi: 10.3389/fnagi.2022.1050715 (PMC9691406; doi:10.3389/fnagi.2022.1050715)
Supplement: Supplementary file 2 [file Table_2.DOCX]

| Table S2. Inconsistency test between direct and indirect treatment comparisons. | | | | | | | | |
| --- | --- | --- | --- | --- | --- | --- | --- | --- |
| Side | Direct | | Indirect | | | Difference | | P>z |
|  | Coefficient | SE | Coefficient | | SE | Coefficient | SE |  |
| AA AK* | 0.172 | 0.409 | -0.426 | 13.156 | | 0.598 | 13.163 | 0.964 |
| AB AL | 1.228 | 0.603 | 0.422 | 0.658 | | 0.806 | 0.893 | 0.367 |
| AB AM* | 1.055 | 0.747 | 0.679 | 200.020 | | 0.376 | 200.021 | 0.999 |
| AB AN | 0.758 | 0.423 | 0.638 | 0.652 | | 0.120 | 0.777 | 0.877 |
| AB AO | 0.495 | 0.628 | 1.586 | 0.693 | | -1.091 | 0.935 | 0.000 |
| AC AL | 1.696 | 0.881 | -0.303 | 0.750 | | 2.000 | 1.156 | 0.084 |
| AC AN | -0.032 | 0.797 | 0.797 | 0.765 | | -0.829 | 1.105 | 0.453 |
| AC AO | 0.029 | 0.934 | 1.121 | 0.794 | | -1.093 | 1.225 | 0.373 |
| AD AN * | 0.412 | 0.733 | 1.409 | 199.991 | | -0.997 | 199.993 | 0.996 |
| AE AF | -0.094 | 0.758 | 2.219 | 1.150 | | -2.313 | 1.377 | 0.093 |
| AE AO | 1.437 | 0.882 | -0.877 | 1.058 | | 2.313 | 1.377 | 0.093 |
| AE AR* | -0.674 | 0.950 | -2.034 | 200.017 | | 1.360 | 200.017 | 0.995 |
| AF AG | -0.233 | 0.772 | 0.469 | 1.327 | | -0.702 | 1.535 | 0.647 |
| AF AV | -1.587 | 0.561 | -0.224 | 0.871 | | -1.363 | 1.036 | 0.188 |
| AF AY | -0.667 | 0.744 | -1.218 | 0.933 | | 0.552 | 1.194 | 0.644 |
| AG AX | -0.968 | 0.868 | -0.265 | 1.266 | | -0.703 | 1.535 | 0.647 |
| AH AL* | 0.363 | 0.736 | 1.734 | 200.031 | | 1.370 | 200.032 | 0.995 |
| AI AL | 0.870 | 0.751 | -0.516 | 1.157 | | 1.386 | 1.379 | 0.315 |
| AI AY | -0.773 | 0.905 | 0.614 | 1.041 | | -1.387 | 1.379 | 0.315 |
| AJ AL * | 0.380 | 0.755 | 1.183 | 89.456 | | -0.803 | 89.46 | 0.993 |
| AJ AP * | -0.259 | 0.792 | -1.597 | 200.073 | | 1.338 | 200.074 | 0.995 |
| AJ BF * | -0.051 | 0.754 | 1.287 | 200.024 | | -1.338 | 200.025 | 0.995 |
| AK AS * | -0.279 | 0.274 | -0.472 | 57.757 | | 0.193 | 57.758 | 0.997 |
| AK AV * | -0.063 | 0.858 | -0.789 | 4.873 | | 0.726 | 14.898 | 0.961 |
| AK AW* | -0.431 | 0.759 | -0.774 | 199.995 | | 0.344 | 199.996 | 0.999 |
| AK BC * | -0.107 | 0.775 | -0.451 | 199.999 | | 0.344 | 200.000 | 0.999 |
| AK BE * | 0.060 | 0.913 | -0.283 | 200.016 | | 0.344 | 200.016 | 0.999 |
| AL AV | -0.604 | 0.803 | -1.206 | 0.709 | | 0.602 | 1.072 | 0.574 |
| AL AX | -0.373 | 0.777 | -1.076 | 1.324 | | 0.703 | 1.535 | 0.647 |
| AL BB | -0.081 | 0.690 | -3.440 | 0.861 | | 3.359 | 1.104 | 0.002 |
| AL BF * | -0.431 | 0.755 | -1.768 | 141.433 | | 1.338 | 141.436 | 0.992 |
| AL BH * | -0.863 | 0.750 | -2.960 | 199.841 | | 2.098 | 199.842 | 0.992 |
| AL BI * | -0.778 | 0.737 | -2.876 | 199.983 | | 2.098 | 199.985 | 0.992 |
| ANAQ* | -0.559 | 0.553 | -2.380 | 141.392 | | 1.821 | 141.393 | 0.990 |
| AN AV | -0.381 | 0.789 | -1.144 | 0.706 | | 0.763 | 1.059 | 0.471 |
| AN AY | -0.268 | 0.854 | -0.717 | 0.830 | | 0.450 | 1.191 | 0.706 |
| AN AZ | -0.091 | 0.451 | 1.012 | 0.865 | | -1.103 | 0.976 | 0.258 |
| AN BB | -1.608 | 0.935 | -1.176 | 0.827 | | -0.433 | 1.248 | 0.729 |
| AN BG * | -0.168 | 0.737 | -1.988 | 0.300 | | 1.820 | 199.977 | 0.993 |
| AO AZ | -0.596 | 0.776 | 0.304 | 0.726 | | -0.900 | 1.062 | 0.397 |
| AS AT * | 0.771 | 0.736 | 0.984 | 199.950 | | -0.214 | 199.951 | 0.999 |
| AS AU* | -0.330 | 0.735 | -0.117 | 200.07 | | -0.214 | 200.071 | 0.999 |
| AT AU | . | . | . | . | | . | . | . |
| AZ BA* | 1.847 | 0.796 | -0.264 | 200.031 | | 2.111 | 200.032 | 0.992 |
| AZ BD | 0.129 | 0.458 | 0.736 | 1.495 | | -5.717 | 1.564 | 0.000 |
| BB BD | 6.148 | 1.338 | 1.736 | 0.810 | | 5.718 | 1.564 | 0.000 |

* All the evidence about these contrasts comes from the trials which directly compare them.

SE: standard error; AA: Bright light therapy; AB: Cognitive behavior therapy; AC: Dance; AD: Deep brain stimulation; AE: Aerobic exercise; AF: Stretching exercise; AG: Balance training; AH: Combin-

ed exercise; AI: Resistance exercise; AJ: Massage; AK: Placebo; AL: Treatment as usual; AM: Clinic- al monitoring; AN: Waitlist; AO: Supportive instruction; AP: Music therapy; AQ: Mindfulness interv- ention; AR: LSVT-BIG therapy; AS: Transcranial magnetic stimulation; AT: Traditional rehabilitation; AU: Repetitive transcranial magnetic stimulation and traditional rehabilitation; AV: Traditional Chine-

se exercise; AW: Transcranial direct current stimulation; AX: Virtual reality; AY: Yoga; AZ: Cognitive training; BA: Occupational group activities; BB: Psychotherapy; BC: Acupuncture; BD: Physiothera-

py; BE: Cognitive behavioral therapy and bright light therapy; BF: Alexander technique; BG: Multidi-

sciplenary rehabilitation; BH: Virtual reality and Wuqinxi; BI: Auricular pressure and pointed psycho-

logical nursing.
