# Supplementary material for: Efficacy of non-pharmacological interventions for depression in individuals with Parkinson's disease: A systematic review and network meta-analysis
Source: Front Aging Neurosci. 2022 Nov 10;14:1050715. doi: 10.3389/fnagi.2022.1050715 (PMC9691406; doi:10.3389/fnagi.2022.1050715)
Supplement: Supplementary file 3 [file Data_Sheet_1.docx]

**PubMed**

(((("Parkinson Disease"[Mesh]) OR ((((((((((((Idiopathic Parkinson's Disease[Title/Abstract]) OR (Lewy Body Parkinson's Disease[Title/Abstract])) OR (Parkinson's Disease, Idiopathic[Title/Abstract])) OR (Parkinson's Disease, Lewy Body[Title/Abstract])) OR (Parkinson Disease, Idiopathic[Title/Abstract])) OR (Parkinson's Disease[Title/Abstract])) OR (Idiopathic Parkinson Disease[Title/Abstract])) OR (Lewy Body Parkinson Disease[Title/Abstract])) OR (Lewy Body Parkinson Disease[Title/Abstract])) OR (Parkinsonism, Primary[Title/Abstract])) OR (Paralysis Agitans[Title/Abstract])) OR (Parkinson*[Title/Abstract]))) AND ((((((((treatment[Title/Abstract] OR intervention[Title/Abstract] OR therapy[Title/Abstract] OR management[Title/Abstract] OR rehabilitation[Title/Abstract]) OR (non-pharmacolog*[Title/Abstract])) OR (non-pharmacological[Title/Abstract])) OR (TMS[Title/Abstract] OR tDCS[Title/Abstract] OR DBS[Title/Abstract] OR taichi[Title/Abstract] OR qigong[Title/Abstract] OR acupuncture[Title/Abstract] OR massage[Title/Abstract] OR song[Title/Abstract] OR music[Title/Abstract] OR dance[Title/Abstract] OR aromatherapy[Title/Abstract] OR moxibustion[Title/Abstract] OR exercise[Title/Abstract] OR CBT[Title/Abstract] OR Psychotherapy[Title/Abstract] OR cognitive training[Title/Abstract] OR Electroconvulsive Therapy[Title/Abstract])) OR (transcranial magnetic stimulation[Title/Abstract])) OR (transcranial Direct Current Stimulation[Title/Abstract])) OR (cognitive behavior training[Title/Abstract])) OR (deep brain stimulation[Title/Abstract]))) AND (("Depression"[Mesh]) OR (((((((Depressive Symptoms[Title/Abstract]) OR (Depressive Symptom[Title/Abstract])) OR (Symptom, Depressive[Title/Abstract])) OR (Symptoms, Depressive[Title/Abstract])) OR (Emotional Depression[Title/Abstract])) OR (Depression, Emotional[Title/Abstract])) OR (depress*[Title/Abstract])))) AND (randomized controlled trial[Publication Type] OR randomized[Title/Abstract] OR placebo[Title/Abstract])

**Cochrane**

#1 MeSH descriptor: [Parkinson Disease] explode all trees

#2 (Idiopathic Parkinson's Disease):ti,ab,kw OR (Parkinson*):ti,ab,kw (Word variations have been searched)

#3 (Lewy Body Parkinson's Disease):ti,ab,kw OR (Parkinson's Disease, Idiopathic):ti,ab,kw OR (Parkinson's Disease, Lewy Body):ti,ab,kw OR (Parkinson Disease, Idiopathic):ti,ab,kw OR (Parkinson's Disease):ti,ab,kw OR (Idiopathic Parkinson Disease):ti,ab,kw OR (Lewy Body Parkinson Disease):ti,ab,kw OR (Primary Parkinsonism):ti,ab,kw OR (Parkinsonism, Primary):ti,ab,kw OR (Paralysis Agitans):ti,ab,kw

#4 #1 OR #2 OR #3

#5 (TMS):ti,ab,kw OR (tDCS):ti,ab,kw OR (DBS):ti,ab,kw OR (taichi):ti,ab,kw OR (qigong):ti,ab,kw OR (acupuncture):ti,ab,kw OR (massage):ti,ab,kw OR (song):ti,ab,kw OR (music):ti,ab,kw OR (dance):ti,ab,kw OR (aromatherapy):ti,ab,kw OR (moxibustion):ti,ab,kw OR (exercise):ti,ab,kw OR (CBT):ti,ab,kw OR (Psychotherapy):ti,ab,kw OR (cognitive training):ti,ab,kw OR (Electroconvulsive Therapy):ti,ab,kw OR (transcranial magnetic stimulation):ti,ab,kw OR (transcranial Direct Current Stimulation):ti,ab,kw OR (cognitive behavior training):ti,ab,kw OR (deep brain stimulation):ti,ab,kw OR (treatment):ti,ab,kw OR (intervention):ti,ab,kw OR (therapy):ti,ab,kw OR (management):ti,ab,kw OR (rehabilitation):ti,ab,kw OR (non-pharmacolog*):ti,ab,kw OR (non-pharmacological):ti,ab,kw

#6 MeSH descriptor: [Depression] explode all trees

#7 (Depressive Symptoms):ti,ab,kw OR (Depressive Symptom):ti,ab,kw OR (Symptom, Depressive):ti,ab,kw OR (Symptoms, Depressive):ti,ab,kw OR (Emotional Depression):ti,ab,kw OR (Depression, Emotional):ti,ab,kw OR (depress*):ti,ab,kw

#8 #6 OR #7

#9 (randomized controlled trial OR randomized OR placebo):pt (Word variations have been searched)

#10 #4 AND #5 AND #8 AND #9

**Embase**

#37. #12 AND #21 AND #35 AND #36

#36. randomized AND controlled AND trial:ab,kw,ti OR randomized:kw,ti OR placebo:ab,kw,ti

#35. #22 OR #23 OR #24 OR #25 OR #26 OR #27 OR #28 OR #29 OR #30 OR #31 OR #32 OR #33 OR #34

#34. depress*:ab,kw,ti

#33. parental AND depression:ab,kw,ti

#32. mental AND depression:ab,kw,ti

#31. depressive AND syndrome:ab,kw,ti

#30. depressive AND symptom:ab,kw,ti

#29. depressive AND state:ab,kw,ti

#28. depressive AND personality AND disorder:ab,kw,ti

#27. depressive AND illness:ab,kw,ti

#26. depressive AND disorder:ab,kw,ti

#25. depressive AND disease:ab,kw,ti

#24. clinical AND depression:ab,kw,ti

#23. ('central'/exp OR central) AND depression:ab,kw,ti

#22. 'depression'/exp

#21. #13 OR #14 OR #15 OR #16 OR #17 OR #18 OR #19 OR #20

#20. deep AND brain AND stimulation:ab,kw,ti

#19. cognitive AND behavior AND training:ab,kw,ti

#18. transcranial AND direct AND current AND stimulation:ab,kw,ti

#17. transcranial AND magnetic AND stimulation:ab,kw,ti

#16. electroconvulsive AND therapy:ab,kw,ti

#15. cognitive AND training:ab,kw,ti

#14. treatment:ab,kw,ti OR intervention:ab,kw,ti OR therapy:ab,kw,ti OR management:ab,kw,ti OR rehabilitation:ab,kw,ti OR 'non pharmacolog*':ab,kw,ti OR 'non pharmacological':ab,kw,ti

#13. tms:ab,kw,ti OR tdcs:ab,kw,ti OR dbs:kw,ab,ti OR taichi:ab,kw,ti OR qigong:ab,kw,ti OR acupuncture:ab,kw,ti OR massage:ab,kw,ti OR song:ab,ti OR music:ab,kw,ti OR dance:ab,kw,ti OR aromatherapy:ab,kw,ti OR moxibustion:ab,kw,ti OR exercise:ab,kw,ti OR cbt:ab,kw,ti OR psychotherapy:kw,ti

#12. #1 OR #2 OR #3 OR #4 OR #5 OR #6 OR #7 OR #8 OR #9 OR #10 OR #11

#11. primary AND parkinsonism:ab,kw,ti

#10. parkinsons AND disease:ab,kw,ti

#9. parkinson AND dementia AND complex:ab,kw,ti

#8. paralysis AND agitans:ab,kw,ti

#7. lewy AND body AND parkinsons AND disease:ab,kw,ti

#6. lewy AND body AND parkinson AND disease:ab,kw,ti

#5. lewy AND bodies AND of AND parkinsons AND disease:ab,kw,ti

#4. lewy AND bodies AND of AND parkinsons AND disease:ab,kw,ti

#3. lewy:ab,kw,ti AND bodies:ab,kw,ti AND of:ab,kw,ti AND parkinson:ab,kw,ti AND disease:ab,kw,ti

#2. parkinson*:ab,kw,ti OR (idiopathic:ab,kw,ti AND parkinsonism:ab,kw,ti)

#1. 'parkinson disease'/exp

**PsycInfo**

1 (Parkinson* or Idiopathic Parkinson's Disease or Lewy Body Parkinson's Disease or Parkinson's Disease, Idiopathic or Parkinson's Disease, Lewy Body or Parkinson Disease, Idiopathic or Parkinson's Disease or Idiopathic Parkinson Disease or Lewy Body Parkinson Disease or Primary Parkinsonism or Parkinsonism, Primary or Paralysis Agitans).ab,ti.

2 exp Parkinson's Disease/

3 1 or 2

4 (TMS or tDCS or DBS, or taichi or qigong or acupuncture or massage or song or music or dance or aromatherapy or moxibustion or exercise or CBT or Psychotherapy or cognitive training or Electroconvulsive Therapy or transcranial magnetic stimulation or transcranial Direct Current Stimulation or cognitive behavior training or deep brain stimulation or treatment or intervention or therapy or management or rehabilitation or non-pharmacolog*).ab,ti.

5 exp "Depression (Emotion)"/

6 (Depressive Symptoms or Depressive Symptom or Symptom, Depressive or Symptoms, Depressive or Emotional Depression or Depression, Emotional or depress*).ab,ti.

7 5 or 6

8 (randomized controlled trial or randomized or placebo).ab,ti.

9 3 and 4 and 7 and 8

**CNKI**

主题 = 帕金森病 OR 主题 = 帕金森 AND 主题 = 非药物治疗 OR 主题 = 康复 OR 主题 = 疗法 OR 主题 = 干预 OR 主题 = 治疗 OR 主题 = 太极 OR 主题 = 气功 OR 主题 = 瑜伽 OR 主题 = 认知训练 OR 主题 = 认知行为疗法 OR 主题 = 经颅磁刺激 OR 主题 = 经颅直流电刺激 OR 主题 = 脑深部电刺激 OR 主题 = 芳香疗法 OR 主题 = 针灸 OR 主题 = 按摩 OR 主题 = 艾灸 OR 主题 = 音乐 OR 主题 = 舞蹈 OR 主题 = 运动 AND 主题 = 抑郁 OR 主题 = 情绪 or ( 题名 = 帕金森病 or 题名 = 帕金森 or 题名 = 非药物治疗 or 题名 = 康复 or 题名 = 疗法 or 题名 = 干预 or 题名 = 治疗 or 题名 = 太极 or 题名 = 气功 or 题名 = 瑜伽 or 题名 = 认知训练 or 题名 = 认知行为疗法 or 题名 = 经颅磁刺激 or 题名 = 经颅直流电刺激 or 题名 = 脑深部电刺激 or 题名 = 芳香疗法 or 题名 = 针灸 or 题名 = 按摩 or 题名 = 艾灸 or 题名 = 音乐 or 题名 = 舞蹈 or 题名 = 运动 or 题名 = 抑郁 or 题名 = 情绪 ) AND ( 摘要=随机 OR 摘要=随机对照 )

**Wanfang**

主题:(帕金森 OR 帕金森病) and 主题:(非药物治疗 OR 康复 OR疗法 OR干预 OR治疗 OR 太极 OR 气功 OR 瑜伽 OR 认知训练 OR 认知行为疗法 OR经颅磁刺激 OR 经颅直流电刺激 OR 脑深部电刺激 OR 芳香疗法 ) and 主题:(抑郁 OR 情绪) and 主题:(随机 OR 随机对照)
